# Ethyl 5-methyl-3-phenyl­isoxazole-4-carboxyl­ate

**DOI:** 10.1107/S160053681301427X

**Published:** 2013-05-31

**Authors:** Chandra, K. Raghu, S. Jeyaseelan, K. B. Umesha, M. Mahendra

**Affiliations:** a Department of Studies in Physics, Manasagangotri, University of Mysore, Mysore 570 006, India; b Department of Chemistry, Yuvaraja’s College, University of Mysore, Mysore 570 005, India; c Department of Physics, St Philomena’s College, Mysore, India

## Abstract

In the title compound, C_13_H_13_NO_3_, the dihedral angle between the phenyl and isoxazole rings is 43.40 (13)°. The eth­oxy­carbonyl group is rotated out of the plane of the isoxazole ring by 16.2 (13)°.

## Related literature

For the biological and pharmacological importance of isoxazoles, see: Lin *et al.* (1997 ▶). For the synthesis of isoxazole derivatives and a related structure, see: Chandra *et al.* (2013 ▶).
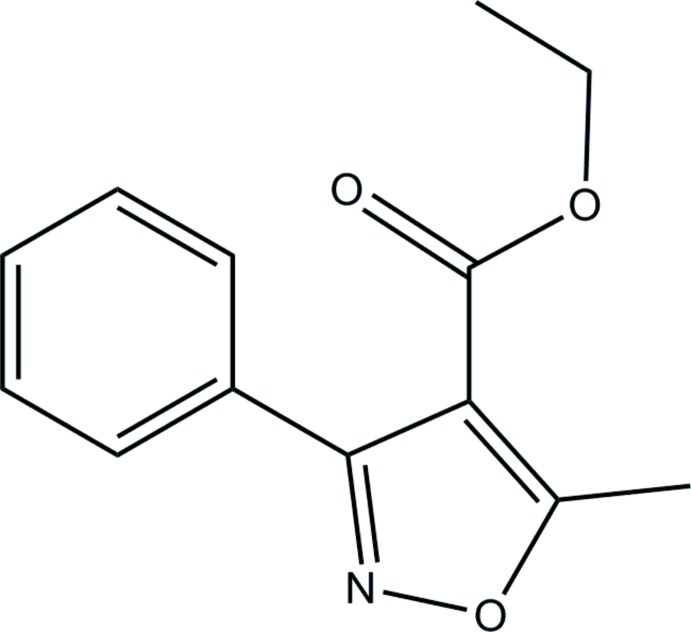


## Experimental

### 

#### Crystal data

C_13_H_13_NO_3_
*M* *_r_* = 231.24
Monoclinic, 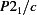

*a* = 9.750 (8) Å
*b* = 14.589 (13) Å
*c* = 9.397 (8) Å
β = 116.872 (13)°
*V* = 1192.3 (18) Å^3^
*Z* = 4
Mo *K*α radiation
μ = 0.09 mm^−1^
*T* = 273 K
0.30 × 0.25 × 0.20 mm

#### Data collection

Bruker APEXII CCD area-detector diffractometer
10036 measured reflections
2060 independent reflections
1340 reflections with *I* > 2σ(*I*)
*R*_int_ = 0.039

#### Refinement

*R*[*F* ^2^ > 2σ(*F* ^2^)] = 0.054
*wR*(*F* ^2^) = 0.186
*S* = 0.99
2060 reflections
156 parameters
H-atom parameters constrained
Δρ_max_ = 0.16 e Å^−3^
Δρ_min_ = −0.17 e Å^−3^

### 

Data collection: *APEX2* (Bruker, 2009 ▶); cell refinement: *SAINT* (Bruker, 2009 ▶); data reduction: *SAINT*; program(s) used to solve structure: *SHELXS97* (Sheldrick, 2008 ▶); program(s) used to refine structure: *SHELXL97* (Sheldrick, 2008 ▶); molecular graphics: *PLATON* (Spek, 2009 ▶); software used to prepare material for publication: *SHELXL97*.

## Supplementary Material

Crystal structure: contains datablock(s) global, I. DOI: 10.1107/S160053681301427X/nc2309sup1.cif

Structure factors: contains datablock(s) I. DOI: 10.1107/S160053681301427X/nc2309Isup2.hkl

Supplementary material file. DOI: 10.1107/S160053681301427X/nc2309Isup3.cml

Additional supplementary materials:  crystallographic information; 3D view; checkCIF report

## Figures and Tables

**Fig. 1.**
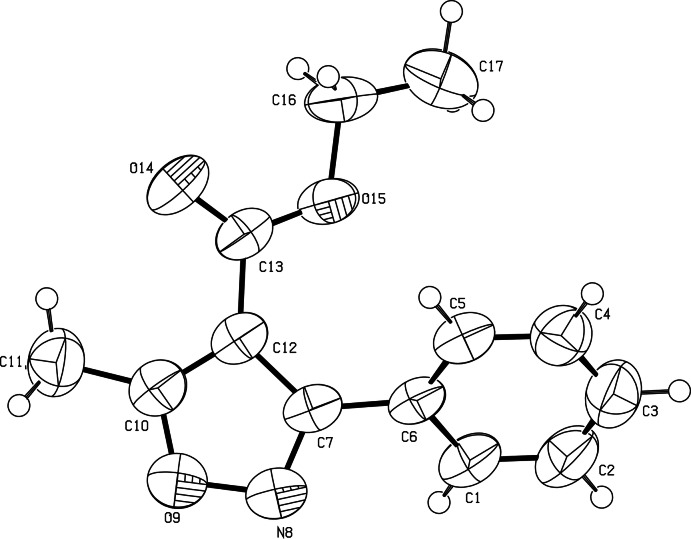
Perspective diagram of the title molecule with labeling and displacement ellipsoids drawn at the 50% probability level.

## supplementary crystallographic information

### Comment

Isoxazole and its derivatives are of well known heterocyclic compounds, which have a variety of biologically activities; such as anti-convulsant, antibacterial, antiasthmatic, and other pharmacological activities (Lin *et al.*, 1997). In view of their importance we have special interest in the synthesis and structural studies of isoxazole derivatives (Chandra *et al.*, 2013). Within this project, the title compound was prepared and characterized by single-crystal X-ray diffraction.

In the molecular structure of the title compound (Fig. 1), the dihedral angle between the phenyl ring (C1/C2/C3/C4/C5/C6) and the isoxazole ring (C7/N8/O9/C10/C12) amount to 43.40 (13)°. The ethoxycarbonyl unit is not in same plane with the isoxazole ring, as indicated by the torsion angle of 16.2 (13)°.

### Experimental

A mixture of benzaldehyde oxime (1 g, 8.33 mmol), chloramine-T (2.33 g, 8.33 mmol) and freshly distilled ethyl acetoacetate (2.16 g, 16.6 mmol) in ethyl alcohol (20 ml) were stirred at 10°C about 6 h. The progress of the reaction was monitored by TLC. After the completion of the reaction the solvent was evaporated in vacuum. The solids thus obtained were recrystalized from hot ethanol to get single crystals of the title compound.

### Refinement

The H atoms were placed in idealized positions and allowed to ride on their parent atoms with C–H distances in the range of 0.93 to 0.97 Å and *U*_iso_(H) = 1.2*U*_eq_(C) (1.5 for methyl H atoms).

### Crystal data

**Table:**

C_13_H_13_NO_3_	*F*(000) = 488
*M**_r_* = 231.24	*D*_x_ = 1.288 Mg m^−^^3^
Monoclinic, *P*2_1_/*c*	Mo *K*α radiation, λ = 0.71073 Å
Hall symbol: -P 2ybc	Cell parameters from 2060 reflections
*a* = 9.750 (8) Å	θ = 2.3–25.0°
*b* = 14.589 (13) Å	µ = 0.09 mm^−^^1^
*c* = 9.397 (8) Å	*T* = 273 K
β = 116.872 (13)°	Block, yellow
*V* = 1192.3 (18) Å^3^	0.30 × 0.25 × 0.20 mm
*Z* = 4	

### Data collection

**Table:**

Bruker APEXII CCD area-detector diffractometer	*R*_int_ = 0.039
ω and φ scans	θ_max_ = 25.0°, θ_min_ = 2.3°
10036 measured reflections	*h* = −11→11
2060 independent reflections	*k* = −17→17
1340 reflections with *I* > 2σ(*I*)	*l* = −11→11

### Refinement

**Table:**

Refinement on *F*^2^	Primary atom site location: structure-invariant direct methods
Least-squares matrix: full	Secondary atom site location: difference Fourier map
*R*[*F*^2^ > 2σ(*F*^2^)] = 0.054	Hydrogen site location: inferred from neighbouring sites
*wR*(*F*^2^) = 0.186	H-atom parameters constrained
*S* = 0.99	*w* = 1/[σ^2^(*F*_o_^2^) + (0.1145*P*)^2^] where *P* = (*F*_o_^2^ + 2*F*_c_^2^)/3
2060 reflections	(Δ/σ)_max_ < 0.001
156 parameters	Δρ_max_ = 0.16 e Å^−^^3^
0 restraints	Δρ_min_ = −0.17 e Å^−^^3^

### Special details

**Table:**

Geometry. Bond distances, angles *etc*. have been calculated using the rounded fractional coordinates. All su's are estimated from the variances of the (full) variance-covariance matrix. The cell e.s.d.'s are taken into account in the estimation of distances, angles and torsion angles
Refinement. Refinement on *F*^2^ for ALL reflections except those flagged by the user for potential systematic errors. Weighted *R*-factors *wR* and all goodnesses of fit *S* are based on *F*^2^, conventional *R*-factors *R* are based on *F*, with *F* set to zero for negative *F*^2^. The observed criterion of *F*^2^ > σ(*F*^2^) is used only for calculating -*R*-factor-obs *etc*. and is not relevant to the choice of reflections for refinement. *R*-factors based on *F*^2^ are statistically about twice as large as those based on *F*, and *R*-factors based on ALL data will be even larger.

### Fractional atomic coordinates and isotropic or equivalent isotropic displacement parameters (Å^2^)

**Table:**

	*x*	*y*	*z*	*U*_iso_*/*U*_eq_	
O9	0.6154 (2)	0.13026 (13)	−0.1802 (3)	0.0780 (8)	
O14	0.6825 (2)	0.12137 (19)	0.2907 (3)	0.1153 (12)	
O15	0.4317 (2)	0.09643 (11)	0.17563 (19)	0.0667 (6)	
N8	0.4554 (3)	0.14345 (15)	−0.2522 (3)	0.0747 (9)	
C1	0.1443 (3)	0.10417 (18)	−0.3147 (3)	0.0721 (10)	
C2	−0.0093 (3)	0.1141 (2)	−0.3603 (4)	0.0840 (11)	
C3	−0.0594 (3)	0.1676 (2)	−0.2736 (4)	0.0861 (11)	
C4	0.0469 (3)	0.21272 (19)	−0.1411 (4)	0.0798 (11)	
C5	0.2008 (3)	0.20332 (16)	−0.0937 (3)	0.0649 (9)	
C6	0.2520 (3)	0.14949 (15)	−0.1799 (3)	0.0571 (8)	
C7	0.4161 (3)	0.13941 (14)	−0.1372 (3)	0.0577 (8)	
C10	0.6666 (3)	0.11924 (16)	−0.0234 (3)	0.0646 (9)	
C11	0.8327 (3)	0.0997 (2)	0.0691 (4)	0.0865 (11)	
C12	0.5479 (3)	0.12501 (14)	0.0123 (3)	0.0592 (8)	
C13	0.5628 (3)	0.11456 (17)	0.1739 (3)	0.0654 (9)	
C16	0.4341 (4)	0.0912 (2)	0.3301 (3)	0.0824 (11)	
C17	0.2752 (4)	0.0730 (2)	0.3043 (4)	0.0957 (14)	
H1	0.17710	0.06700	−0.37380	0.0870*	
H2	−0.08040	0.08420	−0.45110	0.1010*	
H3	−0.16400	0.17340	−0.30390	0.1030*	
H4	0.01350	0.25020	−0.08290	0.0950*	
H5	0.27120	0.23350	−0.00280	0.0780*	
H11A	0.85120	0.03600	0.05850	0.1300*	
H11B	0.86540	0.11380	0.17950	0.1300*	
H11C	0.88940	0.13650	0.02920	0.1300*	
H16A	0.47100	0.14850	0.38720	0.0990*	
H16B	0.50210	0.04240	0.39260	0.0990*	
H17A	0.20880	0.12140	0.24150	0.1430*	
H17B	0.27330	0.06990	0.40550	0.1430*	
H17C	0.24040	0.01570	0.24930	0.1430*	

### Atomic displacement parameters (Å^2^)

**Table:**

	*U*^11^	*U*^22^	*U*^33^	*U*^12^	*U*^13^	*U*^23^
O9	0.0756 (13)	0.0937 (13)	0.0679 (14)	0.0024 (9)	0.0352 (11)	0.0109 (10)
O14	0.0684 (14)	0.196 (3)	0.0521 (14)	−0.0009 (13)	0.0015 (11)	−0.0013 (13)
O15	0.0746 (12)	0.0772 (11)	0.0415 (10)	−0.0015 (8)	0.0203 (9)	0.0048 (7)
N8	0.0768 (16)	0.0898 (15)	0.0555 (15)	0.0044 (11)	0.0281 (12)	0.0099 (11)
C1	0.0728 (18)	0.0765 (16)	0.0501 (16)	−0.0049 (12)	0.0129 (13)	−0.0015 (12)
C2	0.072 (2)	0.091 (2)	0.0633 (19)	−0.0137 (15)	0.0079 (16)	0.0049 (15)
C3	0.0632 (18)	0.091 (2)	0.092 (2)	0.0039 (15)	0.0245 (18)	0.0233 (18)
C4	0.081 (2)	0.0796 (17)	0.076 (2)	0.0125 (14)	0.0329 (16)	0.0104 (15)
C5	0.0744 (17)	0.0609 (14)	0.0497 (15)	0.0009 (11)	0.0195 (13)	0.0038 (11)
C6	0.0634 (15)	0.0558 (12)	0.0411 (14)	−0.0012 (10)	0.0139 (12)	0.0063 (10)
C7	0.0676 (16)	0.0533 (13)	0.0445 (15)	−0.0043 (10)	0.0186 (12)	0.0011 (10)
C10	0.0661 (16)	0.0592 (13)	0.0621 (18)	−0.0047 (11)	0.0234 (14)	0.0014 (11)
C11	0.0643 (18)	0.096 (2)	0.091 (2)	−0.0043 (14)	0.0279 (16)	0.0033 (16)
C12	0.0619 (15)	0.0555 (12)	0.0494 (15)	−0.0033 (10)	0.0157 (12)	0.0027 (10)
C13	0.0633 (17)	0.0723 (15)	0.0466 (16)	0.0026 (12)	0.0126 (13)	0.0014 (11)
C16	0.110 (2)	0.090 (2)	0.0457 (16)	0.0163 (16)	0.0338 (16)	0.0099 (13)
C17	0.121 (3)	0.098 (2)	0.090 (2)	0.0003 (18)	0.067 (2)	−0.0064 (18)

### Geometric parameters (Å, º)

**Table:**

O9—N8	1.405 (4)	C12—C13	1.467 (4)
O9—C10	1.335 (4)	C16—C17	1.480 (6)
O14—C13	1.191 (4)	C1—H1	0.9300
O15—C13	1.313 (4)	C2—H2	0.9300
O15—C16	1.443 (4)	C3—H3	0.9300
N8—C7	1.301 (4)	C4—H4	0.9300
C1—C2	1.367 (5)	C5—H5	0.9300
C1—C6	1.394 (4)	C11—H11A	0.9600
C2—C3	1.368 (5)	C11—H11B	0.9600
C3—C4	1.375 (5)	C11—H11C	0.9600
C4—C5	1.366 (5)	C16—H16A	0.9700
C5—C6	1.374 (4)	C16—H16B	0.9700
C6—C7	1.470 (5)	C17—H17A	0.9600
C7—C12	1.427 (4)	C17—H17B	0.9600
C10—C11	1.479 (5)	C17—H17C	0.9600
C10—C12	1.345 (5)		
			
O14···C2^i^	3.300 (5)	C5···H17A^iii^	3.0100
O14···C11	3.056 (5)	C12···H5	3.0800
O15···C5	2.959 (4)	C13···H11B	2.9300
O15···C10^ii^	3.410 (4)	C13···H5	3.1000
O15···C6	3.089 (4)	C16···H16B^vii^	3.0800
O9···H17C^ii^	2.7900	H1···N8	2.6700
O14···H16B	2.6200	H2···O14^iv^	2.5400
O14···H11B	2.4400	H4···H11C^vi^	2.5500
O14···H16A	2.6300	H4···C1^v^	3.1000
O14···H2^i^	2.5400	H4···C2^v^	2.9600
O15···H5	2.6200	H5···O15	2.6200
N8···H1	2.6700	H5···C12	3.0800
N8···H5^iii^	2.8600	H5···C13	3.1000
C2···O14^iv^	3.300 (5)	H5···N8^v^	2.8600
C5···C17^iii^	3.567 (5)	H11B···O14	2.4400
C5···C13	3.526 (5)	H11B···C13	2.9300
C5···O15	2.959 (4)	H11C···C4^viii^	2.8900
C6···O15	3.089 (4)	H11C···H4^viii^	2.5500
C10···O15^ii^	3.410 (4)	H16A···O14	2.6300
C11···O14	3.056 (5)	H16B···O14	2.6200
C13···C5	3.526 (5)	H16B···C16^vii^	3.0800
C17···C5^v^	3.567 (5)	H16B···H16B^vii^	2.3800
C1···H4^iii^	3.1000	H17A···C5^v^	3.0100
C2···H4^iii^	2.9600	H17C···O9^ii^	2.7900
C4···H11C^vi^	2.8900		
			
N8—O9—C10	109.1 (2)	C1—C2—H2	120.00
C13—O15—C16	116.7 (2)	C3—C2—H2	120.00
O9—N8—C7	105.9 (2)	C2—C3—H3	120.00
C2—C1—C6	120.2 (3)	C4—C3—H3	120.00
C1—C2—C3	120.6 (3)	C3—C4—H4	120.00
C2—C3—C4	119.1 (3)	C5—C4—H4	119.00
C3—C4—C5	121.0 (3)	C4—C5—H5	120.00
C4—C5—C6	120.2 (3)	C6—C5—H5	120.00
C1—C6—C5	118.8 (3)	C10—C11—H11A	109.00
C1—C6—C7	118.7 (2)	C10—C11—H11B	110.00
C5—C6—C7	122.5 (2)	C10—C11—H11C	109.00
N8—C7—C6	117.5 (2)	H11A—C11—H11B	109.00
N8—C7—C12	110.6 (3)	H11A—C11—H11C	109.00
C6—C7—C12	131.9 (3)	H11B—C11—H11C	109.00
O9—C10—C11	115.8 (3)	O15—C16—H16A	110.00
O9—C10—C12	109.5 (3)	O15—C16—H16B	110.00
C11—C10—C12	134.6 (3)	C17—C16—H16A	110.00
C7—C12—C10	104.9 (2)	C17—C16—H16B	110.00
C7—C12—C13	131.2 (3)	H16A—C16—H16B	108.00
C10—C12—C13	123.9 (3)	C16—C17—H17A	110.00
O14—C13—O15	124.0 (3)	C16—C17—H17B	109.00
O14—C13—C12	122.9 (3)	C16—C17—H17C	109.00
O15—C13—C12	113.1 (2)	H17A—C17—H17B	110.00
O15—C16—C17	107.8 (2)	H17A—C17—H17C	109.00
C2—C1—H1	120.00	H17B—C17—H17C	109.00
C6—C1—H1	120.00		
			
C10—O9—N8—C7	−0.4 (3)	C1—C6—C7—N8	42.7 (3)
N8—O9—C10—C11	176.9 (2)	C1—C6—C7—C12	−137.0 (3)
N8—O9—C10—C12	−0.3 (3)	C5—C6—C7—N8	−136.0 (3)
C16—O15—C13—O14	4.6 (4)	C5—C6—C7—C12	44.3 (4)
C16—O15—C13—C12	−175.9 (2)	N8—C7—C12—C10	−1.1 (3)
C13—O15—C16—C17	178.4 (2)	N8—C7—C12—C13	−179.1 (2)
O9—N8—C7—C6	−178.90 (18)	C6—C7—C12—C10	178.7 (2)
O9—N8—C7—C12	0.9 (2)	C6—C7—C12—C13	0.7 (4)
C6—C1—C2—C3	−0.8 (4)	O9—C10—C12—C7	0.8 (3)
C2—C1—C6—C5	0.6 (4)	O9—C10—C12—C13	179.0 (2)
C2—C1—C6—C7	−178.2 (2)	C11—C10—C12—C7	−175.7 (3)
C1—C2—C3—C4	1.1 (5)	C11—C10—C12—C13	2.4 (4)
C2—C3—C4—C5	−1.3 (5)	C7—C12—C13—O14	−164.3 (3)
C3—C4—C5—C6	1.1 (4)	C7—C12—C13—O15	16.2 (3)
C4—C5—C6—C1	−0.7 (4)	C10—C12—C13—O14	18.0 (4)
C4—C5—C6—C7	178.0 (2)	C10—C12—C13—O15	−161.5 (2)

Symmetry codes: (i) *x*+1, *y*, *z*+1; (ii) −*x*+1, −*y*, −*z*; (iii) *x*, −*y*+1/2, *z*−1/2; (iv) *x*−1, *y*, *z*−1; (v) *x*, −*y*+1/2, *z*+1/2; (vi) *x*−1, *y*, *z*; (vii) −*x*+1, −*y*, −*z*+1; (viii) *x*+1, *y*, *z*.
